# Association between television viewing and early childhood overweight and obesity: a pair-matched case-control study in China

**DOI:** 10.1186/s12887-019-1557-9

**Published:** 2019-06-08

**Authors:** Jiajin Hu, Ning Ding, Liu Yang, Yanan Ma, Ming Gao, Deliang Wen

**Affiliations:** 1 Department of Social medicine, School of Public Health, China Medical University, Shenyang, Liaoning China; 2 Curriculum and teaching research office, Research Center of Medical Education, China Medical University, Shenyang, Liaoning China; 3 Department of obstetrics and gynecology, Shenyang Maternity and Child Health Hospital, Shenyang, Liaoning China

**Keywords:** Childhood obesity, Childhood overweight, Case-control study, Television viewing, Risk factors

## Abstract

**Background:**

Television (TV) viewing may affect children’s obesity status. In the present study the association between TV viewing and early childhood overweight/obese status was investigated as well as the association based on age difference.

**Methods:**

The present study included 933 children 1–5 years of age that were individually matched on a 1:2 (cases: controls) ratio based on age and community. Cochran-Mantel-Haenszel test was used to evaluate the association between TV viewing time and the related unhealthy behaviors. Conditional logistic regression models were used to quantify the association between TV viewing and children overweight/obese status in two age groups. The effects of TV viewing-related behaviors on the associations were further investigated.

**Results:**

TV viewing time > 1 h was positively associated with the prevalence of TV viewing-related unhealthy behaviors (*P* < 0.05). After controlling for these behaviors, the association between TV viewing and childhood overweight/obese status was significant among 4- to 5-year-old children (odds ratio, OR = 1.72, 95% confidence interval, CI: 1.16––2.54), but not significant among 1- to 3-year-old children.

**Conclusions:**

Childhood overweight/obese status was positively associated with longer TV viewing time only among 4- to 5-year-old children. The results from the present study may help in identifying the population susceptible for overweight and obesity caused by TV viewing.

**Electronic supplementary material:**

The online version of this article (10.1186/s12887-019-1557-9) contains supplementary material, which is available to authorized users.

## Background

Childhood overweight and obesity have been a serious public health problem in both developing and developed countries [1, 2]. In China, 19.4% of children were obese or overweight by the year 2014, and the rate is predicted to increase to 28% by the year 2030 [3]. Obese children are more likely to develop into obese adults [4], who are more likely to have chronic diseases [5–8] which could increase the mortality in adulthood [9].

The fundamental cause of overweight and obesity is an energy imbalance between calories expended and calories consumed. Television (TV) viewing, as a factor associated with both children’s food intake and physical activity, can affect this balance [10, 11]. In previous research that focused on the association between TV viewing and childhood overweight and obesity, the results were inconsistent. In some studies, a positive association was observed between long TV viewing time and childhood obesity [12–14] and in several studies, a dose-response effect between TV viewing time and childhood body mass index (BMI) was indicated [15, 16]. However, in other studies, an association between TV viewing time and childhood obese status was not observed [17, 18]. In a previous meta-analysis, the association between TV viewing and childhood obesity was stronger among pre-school children [19]; however, the association in early childhood based on age has not been investigated.

Furthermore, in previous studies, the effects of TV viewing on childhood overweight/obese status was considered possibly mediated by unhealthy behaviors related to TV viewing, such as decreasing the time of physical activity, more consumption of unhealthy food, exposure to high-energy food advertisements, unhealthy eating habits, and less sleep duration [20–23], however, the results remained inconsistent. Further evidence should be provided to help illustrate the reasons for the association between TV viewing and childhood overweight and obesity, especially in early childhood when children spend most of their time at home and are more likely to be exposed to the TV environment.

To evaluate the association between early childhood overweight/obese status and TV viewing time, and further investigate age difference in the association, a community-based pair-matched case-control study was conducted in China. The primary hypotheses of this study were the following: 1) more TV viewing time is positively associated with early childhood overweight/obese status; 2) the association between TV viewing time and early childhood overweight/obese status differs depending on the age of children.

## Methods

### Study design and participants

A retrospective pair-matched (caliper matching) case-control study was performed at 15 community health service centers in Shenyang, Liaoning Province. It was reported according to The Strengthening the Reporting of Observational Studies in Epidemiology (STROBE) Statement (see Additional file 1) [24]. In Shenyang, children are covered by a three-tier health care system consisting of approximately 72 community health service centers and 5 district-level Women’s and Children’s Health Centers (also including secondary hospitals), and a city-level Women’s and Children’s Health Center. Each community health service center provides primary health care services for the residents living in the nearby communities and all children are given health examinations at the age of 3, 6, 9, 12, 18, 24, 36, 48, and 60 months at community health service centers. In the present study, 15 centers were randomly selected from the 72 community health service centers and included children who received health examinations from September 2016 to December 2016. The eligibility criteria included children who were 1 to 5 years of age (12–60 months) and were residents of the community covered by the community health services. To improve analysis efficiency, we matched each overweight and obese child with 2 normal-weight children of similar age from the same community health service center. The study sample size was calculated according to the formula of pair-matched case control study. To fit the routine health care examination waves, children under 2 years of age were matched within a 3-month age difference and children 2 to 5 years of age were matched within a 12-month age difference. Research was performed in accordance with the Declaration of Helsinki and the study was approved by the Research and Ethical Committee of China Medical University. Informed written consent was provided by the parents of children who participated in the study.

### Anthropometry

Body weight, length, and height of the children were measured by 2 trained child care physicians at the community health service centers and the average of the 2 measurements were recorded. The child’s length and height were measured using calibrated child stadiometers (Seca 416; Seca Corporation, Hamburg, Germany; Seca 220; Seca Corporation, Hamburg, Germany). The child’s weight was measured using weighing scales (Seca 700; Seca Corporation, Hamburg, Germany). Children were asked to remove their clothes and shoes and also defecate before the measurement. The physician measured children’s weight twice to the nearest 0.01 kg. The children under 3 years of age were measured in the lying position. The children were lying on the midline of the measuring bed floor. The line between the upper edge of both ears and the lower edge of the orbit was perpendicular to the measuring bed floor. The physician held the child’s knees to ensure the lower limbs touch each other and remain close to the measuring bed. The footplate was moved to touch the heels on both sides. The reading was accurate to 0.1 cm. Children over 3 years of age were measured for height. The child stood on the floor of the height meter, eyes facing forward, heels, buttocks, and shoulders resting on the pillar at the same time, and head upright. The physician moved the cursor until it touched the child’s head. The physician’s line of sight was on a horizontal plane with the cursor. The reading was accurate to 0.1 cm [3].

Weight-for-length/weight-for-height was calculated as weight in kilograms divided by length/height in meters (Kg/m). Overweight and obesity were defined as weight-for-length/weight-for-height more than 2 standard deviations (SDs) above the WHO Child Growth Standards median. Normal weight was defined as weight-for-length/weight-for-height between ±2 SDs above WHO Child Growth Standards median.

### Exposure variables

Pediatricians conducted face-to-face interviews with children’s parents using validated standard questionnaires [3]. TV viewing time was reported by the parent after answering the question: How many hours per day did your child spend watching TV in the last 6 months? The child’s physical activity time was reported by the parent after answering the question: How many hours per day did your child engage in sporting activities on average in the last 6 months? Parents were asked what time the child went to bed in the evening and what time the child woke up in the morning. The child’s daytime sleep duration (in hours) was reported by the parents. The child’s total sleep time (in hours) was calculated based on the daytime sleep duration plus night sleep duration. The parents were asked whether their child consumed snacks while watching TV (answer: Yes or No). Parents were asked if their child often watched TV while having meals (answer: Yes or No). Parents were asked if their child often watched advertisements for junk food on TV (answer: Yes or No). In addition, parents were asked to reported the number of times their children had snacks during the last week, including western fast food, fried food, puffed food, ice cream, candy, and sweet beverages.

### Measurement of confounding factors

Several confounding factors were measured according to previous studies [25, 26]. The maternal and paternal educational level was reported by the parents and categorized into 3 groups: secondary school and below, university, and postgraduate. Family income was self-reported by the parents and categorized into 4 groups: < 3000 yuan/month, 3000–5000 yuan/month, 5000–8000 yuan/month, and > 8000 yuan/month. Parents’ height and weight were reported and parental body mass index (BMI) was calculated and divided into 2 groups: overweight and not overweight according to WHO standard (overweight defined as BMI ≥25). Delivery mode was reported by the mother and was categorized into 2 groups: cesarean delivery and vaginal delivery.

### Statistical analyses

Categorical data were analyzed using independent sample chi-square tests and continuous variables were analyzed using independent sample *t*-tests. The association between TV viewing and potential TV viewing-related behaviors in different age groups (1- to 3-year-old children group, 4- to 5-year-old children group) were tested using Cochran-Mantel-Haenszel test. Conditional logistic regression models were used to quantify the association between TV viewing and children’s overweight/obese status in the 2 age groups and further investigate the effects of TV viewing-related behaviors on the associations. The confounders were controlled in a step by step manner. Model 1 controlled for a series of familial characteristic covariates: gender (a dummy variable for males; reference category: females), maternal and paternal educational level (dummy variables for university, postgraduate; reference category: secondary school and below), family income (dummy variables for 3000–5000 RMB yuan/month, 5000–8000 RMB yuan/month, > 8000 RMB yuan/month; reference category: < 3000 RMB yuan/month). Model 2 further controlled for physiological covariates which may affect childhood weight status: maternal and paternal overweight/obese status (a dummy variable for overweight and obese, reference category: not overweight and obese), delivery mode (a dummy variable for cesarean delivery, reference category: vaginal delivery). Model 3 controlled for behavioral covariates which may affect the association between TV watching and child weight status: [25, 26] having snacks while watching TV (a dummy variable for yes, reference category: no), watching TV while having meals (a dummy variable for yes, reference category: no), less sleep duration (a dummy variable for < 10 h/day, reference category: ≥10 h/day), exposure to junk food advertisements (a dummy variable for yes, reference category: no). Odds ratios and 95% confidence intervals (CI) were estimated and all *p*-values were two-tailed, *P* < 0.05 was considered statistically significant.

Several values for the confounding variables were missing, including mother’s educational level (3.8%), father’s educational level (3.2%), family income per month (3.2%), sporting activities of children (9.6%), having snacks while watching TV (0.8%), mother’s overweight status (1.2%), father’s overweight status (1.1%), delivery mode (2.4%), watching TV while eating (1.7%), and watching advertisements for junk food (1.6%). Compared with non-missing value subjects, children with missing values for sporting activities were more likely to watch TV < 1 h/day (*P* < 0.05). Other differences regarding TV watching status or weight status were not found between missing values and non-missing values for children. Several sensitivity analyses were performed. First, BMI z-scores (based on the WHO child growth reference) were used to define children’s overweight/obese status and compared with weight-for-length z-scores based on overweight/obese status. Second, snack eating frequency in the regression models was further adjusted. Third, a minimal sample size of 520 children without any missing values was used in the regression models. To include the entire sample, a multiple imputation technique was used to fill in 10 missing values, which may have been insufficient, based on a previous study. All analyses were performed using Stata S.E. version 13 (Stata Corp, TX, USA). A 2-sided *P*-value < 0.05 was considered statistically significant.

## Results

The present analysis included 311 overweight and obese children and 622 normal weight control subjects. The summary statistics in Table 1 presents the characteristics of control and overweight and obese respondents. Statistical difference in age was not observed between the 2 groups (mean age 3.86 ± 1.17 years in the control group and mean age 3.85 ± 1.18 in the overweight and obese group). Gender difference was not observed between the 2 groups (63.7% of controls were males and 65.6% of overweight and obese subjects were males). The parents of overweight and obese children were more likely to be overweight than parents of normal weight children (rate of overweight: 35.7% for the mother; rate of overweight: 76.3% for the father, *P* < 0.001). Overweight and obese children were more likely to watch TV longer (*P* = 0.001). Time spent in sporting activities was not statistically significantly different in the 2 groups. In terms of TV viewing-related behaviors, overweight and obese children had a higher rate of eating more snacks while watching TV (*P* = 0.01) and watching TV while eating (*P* = 0.001).

**Table 1:** Characteristics of study participants: comparison of overweight and obese cases with controls

Characteristics	Controls (*n* = 622)	Cases (*n* = 311)	*P*-value^a^
Age of child (years), M (SD)	3.86 (1.17)	3.85 (1.18)	0.84
Gender, n (%)			0.56
Male	3956 (63.7)	204 (65.6)	
Female	226 (36.3)	107 (34.4)	
Mother’s educational level, n (%)			0.79
Secondary school and below	195 (31.4%)	91 (29.3%)	
University	362 (60.5%)	188 (62.7%)	
Postgraduate	41 (6.9%)	21 (7.0%)	
Father’s educational level, n (%)			0.92
Secondary school and below	195 (32.3%)	100 (33.4%)	
University	359 (59.4%)	176 (58.9%)	
Postgraduate	50 (8.3%)	23 (7.7%)	
Family income per month (yuan), n (%)			0.16
< 3000	53 (8.8%)	26 (8.7%)	
3000–5000	151 (25.0%)	86 (28.7%)	
5000–8000	209 (34.7%)	82 (27.3%)	
> 8000	190 (31.5%)	106 (35.3%)	
Mother is overweight, n (%)			< 0.001
No	491 (80.0%)	198 (64.63%)	
Yes	123 (20.0%)	110 (35.7%)	
Father is overweight, n (%)			< 0.001
No	272 (44.2%)	73 (23.7%)	
Yes	343 (55.8%)	235 (76.3%)	
Cesarean delivery, n (%)			0.03
No	202 (33.1%)	78 (25.9%)	
Yes	408 (66.9%)	233 (74.1%)	
TV viewing time, n (%)			0.001
< 1 h	265 (42.6%)	97 (31.2%)	
≥ 1 h	357 (57.4%)	214 (68.8%)	
Time spent in sporting activities, n (%)			0.27
≤ 1 h	288 (51.9%)	161 (55.9%)	
> 1 h	267 (48.1%)	127 (44.1%)	
Sleep duration, n (%)			0.349
< 10 h	137 (22.0%)	77 (24.8%)	
≥ 10 h	485 (78.0%)	234 (75.2%)	
Having snacks while watching TV, n (%)			0.01
No	394 (63.9%)	169 (54.7%)	
Yes	223 (36.1%)	140 (45.3%)	
Watching TV while having meals, n (%)			0.001
No	385 (62.9%)	158 (51.8%)	
Yes	227 (37.1%)	147 (48.2%)	
Advertisement for junk food, n (%)			0.13
No	413 (67.4%)	190 (62.3%)	
Yes	200 (32.6%)	115 (37.7%)	

*TV* television, *M* mean, *SD* standard deviation

^a^analysis of group differences using *t*-test or chi-square test

Table 2 shows the association between TV viewing time and potential TV viewing-related behaviors based on children’s age. In both age groups, watching TV > 1 h per day was positively associated with the behavior of watching TV while having meals, having snacks while watching TV, and exposure to advertisements for junk food. Time spent in sporting activities was only associated with TV viewing time among the 4- to 5-year-old children; however children who watched TV > 1 h per day were more likely to engage in sporting activities > 1 h per day.

**Table 2:** Association between TV viewing time and TV viewing-related behaviors based on age groups

	TV viewing time							
	1-to 3-year-old children				4- to 5-year-old children			
	< 1 h	≥1 h	OR	95% CI^a^	< 1 h	≥1 h	OR	95% CI^a^
Time spent in sporting activities, n (%)			1.181	0.721–1.932			1.688^b^	1.219–2.336
≤ 1 h	51 (52.0)	71 (50.4)			166 (66.4)	226 (53.9)		
> 1 h	47 (42.0)	70 (46.1)			84 (33.6)	193 (46.1)		
Sleep duration, n (%)			0.953	0.436–2.085			0.964	0.679–1.370
< 10 h	12 (10.7)	17 (11.2)			68 (27.2)	117 (27.9)		
≥ 10 h	100 (89.3)	135 (88.8)			182 (72.8)	302 (72.1)		
Watching TV while having meals, n (%)			3.774^b^	2.194–6.490			4.013^b^	2.798–5.755
No	86 (76.8)	71 (46.7)			198 (79.2)	204 (48.7)		
Yes	26 (23.2)	81 (53.3)			52 (20.8)	215 (51.3)		
Having snacks while watching TV, n (%)			5.844^b^	3.152–10.836			3.347^b^	2.361–4.746
No	96 (85.7)	77 (50.7)			191 (76.4)	206 (49.2)		
Yes	16 (14.3)	75 (49.3)			59 (23.6)	213 (50.8)		
Advertisement for junk food, n (%)			3.697^b^	2.031–6.727			1.810^b^	1.287–2.547
No	94 (83.9)	89 (58.6)			183 (73.2)	252 (60.1)		
Yes	18 (16.1)	63 (41.4)			67 (26.8)	167 (39.9)		

*TV* television, *OR* odds ratio, *CI* confidence interval

^a^CI and OR were obtained using Cochran-Mantel-Haenszel test analysis

^b^Statistically significant at 0.01 level

Table 3 shows that after controlling for family covariates and biological covariates (model 2), TV viewing time was positively associated with children’s overweight/obese status among the 4- to 5-year-old children (OR = 1.81 95% CI: 1.26–2.58). When further controlling for TV viewing-related behaviors (model 3), the trends remained the same, however, the OR decreased to 1.72 (95% CI, 1.16–2.54). The influence of TV viewing on childhood overweight/obese status among 1- to 3-year-old children was not statistically significant in all models. In sensitivity analyses, the results remained consistent when further adjusted for snack eating frequency, or defining overweight/obesity based on BMI z-score, or using the non-missing value samples (Additional file 2: Table S1-S3).

**Table 3:** Adjusted OR and 95% CI for the effects of TV viewing time on childhood overweight/obese status based on age groups

	Overweight/obese status					
	1-to 3-year-old children			4- to 5-year-old children		
TV viewing ≥1 h per day	OR	95% CI	*p*-value^a^	OR	95% CI	*p*-value^a^
Model 1^b^	1.51	0.87–2.62	0.14	1.81	1.26–2.58	0.001
Model 2^c^	1.48	0.84–2.59	0.18	1.90	1.31–2.74	0.001
Model 3^d^	1.20	0.64–2.24	0.57	1.72	1.16–2.54	0.007

*TV* television, *OR* odds ratio, *CI* confidence interval

^a^CI and OR were obtained using conditional logistic regression model analysis

^b^Adjusted for gender, maternal educational level, paternal educational level, family income

^c^Model 1 + maternal weight status, paternal weight status, delivery mode

^d^Model 2 + time spent by children in sporting activities, sleep duration of children, watching television while having meals, having snacks while watching television, exposure to advertisements for junk food

## Discussion

In the present study, the association between TV viewing and overweight/obese status was examined among Chinese preschool children. To the best of our knowledge, this is the first pair-matched case-control study the relationship between TV viewing and childhood overweight/obese status was investigated. The results from the study indicate that early childhood overweight/obese status was positively associated with longer TV viewing time. However, the association existed only among the 4- to 5-year old children. Longer TV viewing time was associated with TV viewing-related unhealthy behaviors among both 1- to 3-year-old and 4- to 5-year-old children.

The results from the current study indicate the effects of TV viewing on childhood overweight/obese status differed among children in different age groups. The 4–5-year-old children had higher risk to be overweight and obese if they watched TV > 1 h per day. This finding is consistent with previous studies. For example, Koleilat et al. found that among 3- to 4-year-old children, watching TV or videos > 1 h a day was independently related to the odds of being obese [27]. Manios et al. found the prevalence of obesity was significantly higher among children who watched TV ≥2 h per day compared with children who watched TV < 2 h per day [25]. The present study results indicate that among 1- to 3-year-old-children, the effects of TV viewing time on overweight/obese status was not statistically significant before or after adjusting for potential covariates, which is partly consistent with the findings of Manios and his colleagues showing that after adjusting for children’s energy intake, no association was found between children overweight/obese status and TV viewing time [25].

Historically, the potential reasons for the association between TV viewing and obesity has been determined as follows: 1) TV viewing may shape unhealthy dietary habits and increase the total energy intake; 2) TV viewing may lead to exposure to unhealthy food advertisements; 3) more TV viewing time may reduce physical activities and increase the amount of sedentary activity; 4) TV viewing may reduce sleep duration [19, 20, 28]. The results from the present study partially confirm these reasons. In 1- to 3-year-old-children, having snacks while watching TV was associated with childhood overweight/obese status, and watching TV while having meals was associated with 4- to 5-year-old-children’s overweight/obese status. In both age groups, watching TV > 1 h per day was positively associated with having snacks while watching TV and having meals while watching TV. Similar results were found by Harris and colleagues [29] who showed watching TV increases snack eating by 45% and snacking while watching TV for 30 min per day leads to a 10 pound weight gain per year for children. Children who watched TV > 1 h per day were more likely to be exposed to unhealthy food advertisements based on results from the present study. Advertisements can influence children’s food preferences and purchase requests directed at parents [30]. Children exposed to advertisements requested significantly more junk food than their counterparts [31], which can lead to childhood obesity. The results from the present study indicated TV viewing time was not associated with the amount of time spent in sporting activities for 1-to 3-year-old children but positively associated with 4- to 5-year-old-children. Therefore, physical activity may not be the most likely reason for the relationship between obesity and TV viewing among preschoolers, which is in agreement with several previous studies [32–34]. However, after adjusting for these covariates, the effects of TV viewing on childhood overweight/obese status remained, indicating the existence of other reasons for the association.

The present study had several limitations. First, the case and control pairs could not be matched based on gender due to the limited sample size, however, statistical gender differences were not found between case and control groups. Second, TV viewing time and sporting activity were reported by the parents, which may lead to measurement and memory biases. Third, due to the limitation in variables investigated and study design, the reasons underlying the association could not be further explained or the causal relationship between TV viewing and childhood overweight/obese status proved. Fourth, residual confounding factors such as children’s energy intake and physical activity level during leisure time exist, which were not accounted for in this analysis.

## Conclusions

The results from the present study indicate that early childhood overweight/obese status was positively associated with longer TV viewing time. However, the association was only statistically significant among the 4- to 5-year-old children. The findings from the present study can help identify the population susceptible to become overweight/obese due to TV viewing and implement the intervention strategy. Watching TV may cause children to become overweight/obese by increasing the exposure to unhealthy food advertisements or shaping unhealthy dietary habits. Future cohort studies should be conducted to further prove the causal relationship found in the present study.

## Additional files

Additional file 1: STROBE 2007 (v4) checklist of items to be included in reports of observational studies in epidemiology* Checklist for cohort, case-control, and cross-sectional studies (combined). (DOC 97 kb)

Additional file 2: **Table S1.** Association between TV viewing time and childhood overweight/obese status after adjusting child snack having frequency. **Table S2.** Association between TV viewing time and childhood overweight/obese status based on WHO BMI z-score standard. **Table S3.** Association between TV viewing time and childhood overweight/obese status among 520 non-missing value samples. (DOCX 19 kb)

## Data Availability

The datasets used and analyzed during the current study are available from the corresponding author upon request.

## Abbreviations

BMI: Body mass index
TV: Television
WHO: World Health Organization
