# Supplementary material for: Association between television viewing and early childhood overweight and obesity: a pair-matched case-control study in China
Source: BMC Pediatr. 2019 Jun 8;19:184. doi: 10.1186/s12887-019-1557-9 (PMC6556004; doi:10.1186/s12887-019-1557-9)
Supplement: Supplementary file 2 — Table S1. Association between TV viewing time and childhood overweight/obese status after adjusting child snack having frequency. Table S2. Association between TV viewing time and childhood overweight/obese status based on WHO BMI z-score standard. Table S3. Association between TV viewing time and childhood overweight/obese status among 520 non-missing value samples. (DOCX 19 kb) [file 12887_2019_1557_MOESM2_ESM.docx]

**Table S1. Association between TV viewing time and childhood overweight/obese status after adjusting child snack having frequency**

|  | **Overweight/obese status** | | | | | |
| --- | --- | --- | --- | --- | --- | --- |
|  | **1-to 3-year-old children** | | | **4- to 5-year-old children** | | |
| **TV viewing ≥ 1 hour per day** | **OR** | **95%CI** | ***p*-value^a^** | **OR** | **95%CI** | ***p*-value^a^** |
| **Model 1^b^** | 1.18 | 0.63-2.21 | 0.61 | 1.71 | 1.16-2.54 | **0.01** |

TV, television; OR, odds ratio; CI, confidence interval

^a^ CI and OR were obtained using conditional logistic regression model analysis

^b^ Adjusted for gender, maternal educational level, paternal educational level, family income, maternal weight status, paternal weight status, delivery mode, time spent by children in sporting activities, sleep duration of children, watching television while having meals, having snacks while watching television, exposure to advertisements for junk food, child snack having frequency.

**Table S2. Association between TV viewing time and childhood overweight/obese status based on WHO BMI z-score standard**

|  | **Overweight/obese status** | | | | | |
| --- | --- | --- | --- | --- | --- | --- |
|  | **1-to 3-year-old children** | | | **4- to 5-year-old children** | | |
| **TV viewing ≥ 1 hour per day** | **OR** | **95%CI** | ***p*-value^a^** | **OR** | **95%CI** | ***p*-value^a^** |
| **Model 1^b^** | 1.35 | 0.81-2.24 | 0.25 | 1.68 | 1.21-2.33 | **0.002** |
| **Model 2^c^** | 1.34 | 0.79-2.28 | 0.27 | 1.74 | 1.23-2.44 | **0.002** |
| **Model 3^d^** | 1.12 | 0.62-2.01 | 0.71 | 1.59 | 1.11-2.29 | **0.01** |

BMI: body mass index; WHO: World Health Organization; TV: television; OR, odds ratio; CI, confidence interval

^a^ CI and OR were obtained using conditional logistic regression model analysis

^b^ Adjusted for gender, maternal educational level, paternal educational level, family income

^c^ Model 1 + maternal weight status, paternal weight status, delivery mode

^d^ Model 2 + time spent by children in sporting activities, sleep duration of children, watching television while having meals, having snacks while watching television, exposure to advertisements for junk food

**Table S3. Association between TV viewing time and childhood overweight/obese status among 520 non-missing value samples**

|  | **Overweight/obese status** | | | | | |
| --- | --- | --- | --- | --- | --- | --- |
|  | **1-to 3-year-old children** | | | **4- to 5-year-old children** | | |
| **TV viewing ≥ 1 hour per day** | **OR** | **95%CI** | ***p*-value^a^** | **OR** | **95%CI** | ***p*-value^a^** |
| **Model 1^b^** | 1.52 | 0.86-2.69 | 0.15 | 1.80 | 1.24-2.60 | **0.002** |
| **Model 2^c^** | 1.47 | 0.81-2.67 | 0.21 | 2.01 | 1.35-2.98 | **0.001** |
| **Model 3^d^** | 1.05 | 0.50-2.20 | 0.89 | 1.91 | 1.23-2.98 | **0.004** |

TV: television; OR, odds ratio; CI, confidence interval

^a^ CI and OR were obtained using conditional logistic regression model analysis

^b^ Adjusted for gender, maternal educational level, paternal educational level, family income

^c^ Model 1 + maternal weight status, paternal weight status, delivery mode

^d^ Model 2 + time spent by children in sporting activities, sleep duration of children, watching television while having meals, having snacks while watching television, exposure to advertisements for junk food
